# Sleep and Cognition: Results From a Longitudinal Cohort of Older Puerto Rican Adults

**DOI:** 10.1093/geroni/igab046.290

**Published:** 2021-12-17

**Authors:** Sandra Arevalo

**Affiliations:** California State University, Torrance California, United States

## Abstract

We examined cross-sectional and prospective associations of sleep duration and insomnia symptoms with measures of cognitive function among older adults aged 45-75 y from the Boston Puerto Rican Health Study, a longitudinal cohort of 1500 participants of Puerto Rican ancestry. We found, statistically significant cross-sectional associations of sleep duration (hours) and an executive function domain before (F=6.20; Prob>F=0.0001) and after (F=2.33; Prob>F=0.05) controlling for covariates (age, sex, education, smoking, drinking, mental and health conditions and medication use); between sleep duration and global cognition before (F=5.38; Prob>F=0.0003) and a trend after controlling for covariates (F=2.20; Prob>F=0.0669). In longitudinal associations, sleep duration (time2) was significantly associated with global condition at time3 (F=2.42; Prob>F=0.0475) after controlling for time2 global cognition. In conclusion, we found hours of sleep and insomnia symptoms significantly associated with various cognitive factors. A public health focus on sleep hygiene may improve cognitive health outcomes in older Puerto Rican adults.

